# Isometric blood flow restriction exercise: acute physiological and neuromuscular responses

**DOI:** 10.1186/s13102-021-00239-7

**Published:** 2021-02-12

**Authors:** Benedikt Lauber, Daniel König, Albert Gollhofer, Christoph Centner

**Affiliations:** 1 Department of Neurosciences and Movement Sciences, University of Fribourg, Fribourg, Switzerland; 2 Department of Sport and Sport Science, University of Freiburg, Schwarzwaldstraße 175, 79117 Freiburg, Germany; 3 Praxisklinik Rennbahn, Muttenz, Switzerland

**Keywords:** Blood flow restriction, Isometric, Metabolic stress, Myoelectric activity, Electromyography, Muscle swelling

## Abstract

**Background:**

Numerous studies have demonstrated that the addition of blood flow restriction (BFR) to low-load (LL) resistance exercise leads to elevated levels of muscle hypertrophy and strength gains. In terms of main underlying mechanisms, metabolic accumulation and increased neuromuscular adaptations seem to play a primary role. However, this evidence is largely based on dynamic exercise conditions. Therefore, the main objective was to investigate the acute physiological adaptations following isometric LL-BFR exercise.

**Methods:**

Fifteen males participated in this cross-over trial and completed the following sessions in a random and counterbalanced order: isometric LL-BFR exercise (20% maximum voluntary contraction, MVC) and load matched LL exercise without BFR. Lactate levels, muscle activation as well as muscle swelling were recorded during the whole exercise and until 15 min post completion. Additionally, changes in maximal voluntary torque and ratings of perceived exertion (RPE) were monitored.

**Results:**

During exercise, EMG amplitudes (72.5 ± 12.7% vs. 46.3 ± 6.7% of maximal EMG activity), muscle swelling and RPE were significantly higher during LL-BFR compared to LL (*p* < 0.05). Lactate levels did not show significant group differences during exercise but revealed higher increases 15 min after completion in the LL-BFR condition (LL-BFR: + 69%, LL: + 22%) (*p* < 0.05). Additionally, MVC torque significantly decreased immediately post exercise only in LL-BFR (~ − 11%) (p < 0.05) but recovered after 15 min.

**Conclusions:**

The present results demonstrate that isometric LL-BFR causes increased metabolic, neuromuscular as well as perceptual responses compared to LL alone. These adaptations are similar to dynamic exercise and therefore LL-BFR represents a valuable type of exercise where large joint movements are contraindicated (e.g. rehabilitation after orthopedic injuries).

## Background

During the last two decades, research has coherently demonstrated that the induction of local hypoxia augments the adaptive responses of human muscles following low-load (LL) (20–40% one repetition maximum, 1RM) resistance training [1]. Although positive adaptations following blood flow restriction (BFR) training were seen for structural (such as muscle mass [2]) as well as and functional outcomes like muscle strength [3], rate of torque development [4] and sprint performance [5], the underlying mechanisms behind these adaptations are not fully understood.

Metabolic stress has been suggested to play a key role [6] by mediating further secondary factors such as an elevated growth hormone (GH) secretion [7], increased fiber type II recruitment [8], muscle cell swelling [9] and the production of reactive oxygen species [10].

Although GH’s effect on skeletal muscle size and performance is questionable [11–13], a significant increase in growth hormone secretion has been consistently shown during high levels of metabolic stress, such as when using BFR.

Surprisingly, these underlying physiological mechanisms have predominantly been investigated under dynamic contraction modes [7, 14–17] although the advantages of LL-BFR training come into play when prescribed during rehabilitation [18] or training of older individuals [1]. In these settings, isometric contraction modes are often preferred over dynamic exercises in the very early phase of rehabilitation where joint movements are often limited [19, 20].

While dynamic exercise seems to be superior to isometric exercise for strength and hypertrophy [21], there is evidence that isometric exercise can increase strength within a limited range of motion specific to that in which the isometric training was performed [22]. Additionally, evidence suggests that also tendon blood volume as well as tendon stiffness adaptations differ between both contraction modes (dynamic vs. isometric) [23]. Regarding muscular adaptations, ambiguous results have been identified with one study showing more pronounced muscle adaptations with dynamic resistance training [24] and others highlighting superior effects of isometric resistance training [25].

Against the background of the ambiguous results and the lack of studies investigating LL-BFR training under isometric conditions, the purpose of this study was to investigate the effects of an acute session of isometric resistance exercise with and without LL-BFR. Because LL-BFR was shown to influence muscle mass [2], neural activation [26] as well as metabolic accumulation [7], the present study combined several physiological measures evaluating potential changes in these parameters. Thus, we want to obtain a detailed understanding of the involved physiological mechanisms which may lead to optimized training programs and further help to improve outcomes in a clinical or athletic setting.

## Methods

### Subjects

Fifteen young men (26 ± 2 years) participated in this study. All subjects were healthy and recreationally active (2–3 h physical activity per week). Inclusion criteria were: age between 18 and 35 years, experience in resistance training (> 1 year), non-smoker, no neurological, acute orthopedic injuries as well as chronic diseases and no history of deep vein thrombosis. Additionally, participants with acute lower extremity injuries and uncontrolled hypertension were excluded from the study.

Approval of the study was obtained by the local ethics committee and all procedures were in accordance with the latest revision of the Declaration of Helsinki. All participants were informed about the potential risks before written informed consent was given.

### Study design

To investigate the effects of BFR on muscle excitation, metabolic accumulation, muscle cell swelling and recovery, a repeated measures cross-over design was implemented. One week before start of the study, participants underwent a preliminary screening which included a medical anamnesis as well as an examination to confirm the compliance with the abovementioned inclusion criteria. After eligibility, all fifteen participants completed two isometric resistance exercise sessions of the knee extensors on two different days in a random and counterbalanced order: 1) low-load (20% MVC) resistance exercise under normal blood flow conditions and 2) low-load (20% MVC) resistance exercise with simultaneous BFR. Prior to measurements, all participants were instructed to avoid strenuous and unaccustomed exercise for 72 h and to follow a 12 h fasting period. All sessions were performed at the same time of the day (between 8 a.m. and 12 a.m.). To ensure an adequate wash-out and recovery period, both sessions were separated by a minimum of 7 and a maximum of 10 days. All outcome assessors were blinded to the respective exercise session.

### Procedures

#### Exercise protocols

##### Low-load resistance exercise (LL)

During this session, low-load resistance exercise was performed at 20% of each individual’s maximum voluntary torque. The subjects were sitting in front of a computer screen watching a red line representing the torque produced by the subjects. This line had to be matched with a black line representing the individual 20% MVC torque. The subjects completed three sets of 90 s of isometric knee extension (knee angle 90°). A resting period of 30 s was allowed between each set. A rest period of 30s was chosen since findings from a previous meta-analysis have demonstrated that lower resting periods seem to augment certain muscular adaptations (e.g. muscle strength) compared to longer resting periods in LL-BFR training [27].

##### Low-load resistance exercise with blood flow restriction (LL-BFR)

During this condition, participants performed the same exercise but had a 12 cm pneumatic nylon cuff [Tourniquet Touch TT20, VBM Medizintechnik GmbH, Germany] applied around the most proximal portion of both thighs. Before starting the session, arterial occlusion pressure (AOP) was determined in a sitting position for each participant. The cuff pressure was steadily increased until the arterial pulse at the posterior tibial artery was no longer detected by Doppler ultrasound [Handydop, Kranzbühler, Solingen, Germany]. This point was defined as 100% of arterial occlusion. During exercise, cuff pressure was preset to 50% of each individual’s AOP and kept inflated during the entire session including the 30 s interset rest periods. This pressure was chosen to be able to compare with previous studies being conducted under dynamic contraction modes [10, 28].

### Dependent variables

#### Muscle activation

Before the electrodes were attached to the vastus lateralis (VL), the skin of the subjects was shaved and cleaned with disinfectant. Electromyographic muscle activation (EMG) was assessed using biopolar surface electrodes [Blue sensor P, Ambu, Bad Nauheim, Germany] at the VL at 50% of femur length (from greater trochanter to the inferior border of the lateral epicondyle) and 2 cm interelectrode distance. The axis of both electrodes was aligned with the muscle fiber orientation. A reference electrode was placed on the patella, and all signals were pre-amplified (1000 ×), band-pass filtered (10–1000 Hz) and sampled at 2048 Hz using a TMSi refa system (TMSi, Twente, The Netherlands). Additionally, electrode positions were marked with a surgical pen in order to replicate the exact locations during the subsequent session. Maximal muscle activity was obtained during the initial three MVCs and the highest rectified EMG value obtained in the MVCs was taken as the maximal EMG activation used for normalizing the EMG data during the subsequent 90s intervals. The VL activation during the 90s intervals was determined by calculating the root mean square (RMS) of the rectified EMG of 10 s intervals.

##### Metabolic accumulation

Metabolic accumulation was estimated via lactate concentration levels. Before the exercise session, following each of the three sets as well as 15 min post completion, 20 μl of capillary blood were obtained from the ear lobe. All samples were analyzed via enzymatic-amperometric methods using a Biosen S-Line lactate analyzer from EKF Diagnostics [Cardiff, UK].

##### Muscle cell swelling

Muscle cell swelling was calculated by measuring acute changes in muscle thickness using b-mode ultrasound. Muscle thickness of the rectus femoris (RF) muscle was measured at 50% of femur length before the exercise session, immediately after each set as well as following 15 min after completion. An ultrasound device [8 MHz, ArtUs EXT-1H; Telemed, Vilnius, Lithuania] with a 60 mm linear transducer was used to acquire sagittal images at the mid distance between the medial-lateral boarders of RF. During the measurement, participants were positioned in a sitting position with their knee and hip angles at 90°. The baseline picture was acquired after a resting period of 20 min, which was implemented to allow fluid shifts. During the whole procedure, participants were instructed to relax their muscle as much as possible. Additionally, a sufficient amount of ultrasound gel was used in order avoid pressure to the skin causing muscle compression. This was ensured by confirming a clearly visible ultrasound gel layer on each image. During each time point, three images were obtained.

For offline analyses, the shortest distance between upper and lower aponeurosis was measured at 25, 50 and 75% of each image. Each image was analyzed three times and the mean of all distances and images was used for further statistical analyses. Reliability of the ultrasound image analyses was confirmed by a very low coefficient of variation of 1.46%.

##### Decrements in maximum voluntary torque

Unilateral, isometric maximum voluntary contraction (MVC) torque at 90° knee extension was measured before, immediately after as well as 15 min following completion of the exercise session using an isokinetic dynamometer [ISOMED 2000, Ferstl, Germany]. Subjects were placed in supine position with restricted shoulders and hips. During the entire procedure knee and hips were fully extended. In total, three trials were conducted with a rest period of 1 min. The mean of all three trials was used for data analysis. All data were normalized to body weight.

##### Perceptual responses

To measure the rating of perceived exertion (RPE), a conventional BORG scale [29] was utilized. Participants were instructed to rate their current RPE on a rating scale reaching from 6 ‘very, very light’ to 20 ‘very, very hard’. RPE was rated before the exercise session, following each of the three sets as well as 15 min after completion.

##### Statistics

Normal distribution and homogeneity of variances was checked for all variables. To test for difference in the EMG during the MVCs between the sets 1–3, a repeated measures ANOVA (rmANOVA) was calculated with the factors time (Pre, Post 15) and condition (LL-BFR, LL). Differences in EMG between the sets were tested by a rmANOVA with factors time (Set 1, Set 2, Set 3) and condition (LL-BFR, LL). For changes in MVC torque, a rmANOVA with factors time (Pre, Set 3, Post 15) and condition (LL-BFR, LL) was conducted.

For all other variables, individual rmANOVAs with factors time (Pre, Set 1, Set 2, Set 3, Post 15) and condition (LL-BFR, LL) were calculated. This included EMG during the 90s intervals, muscle thickness, lactate concentrations and RPE. In case of significant interactions effects, an analysis of simple effects was conducted.

Software package SPSS 24.0 [IBM, Armonk, USA] was used for all statistical analyses. Data is presented as mean ± standard deviation, if not indicated otherwise. The level of significance was set to *p* < 0.05 for all tests.

## Results

All *N* = 15 participants completed both exercise sessions without the occurrence of any adverse events. Anthropometric characteristics are presented in Table 1. No significant between condition differences in main outcomes were observed at baseline.

**Table 1:** Participant characteristics (*N* = 15)

Variable	Mean ± SD	MIN	MAX
Age (years)	25.7 ± 2.3	21	29
Height (cm)	180.1 ± 6.4	169	192
Weight (kg)	74.5 ± 6.9	61.0	87.9
BMI (kg/m^2^)	22.9 ± 1.24	21.4	25.3
AOP (mmHg)	210.0 ± 12.2	180	230

*Note: BMI*   Body Mass Index, *AOP*   Arterial Occlusion Pressure

### Muscle activation

**Fig. 1:**
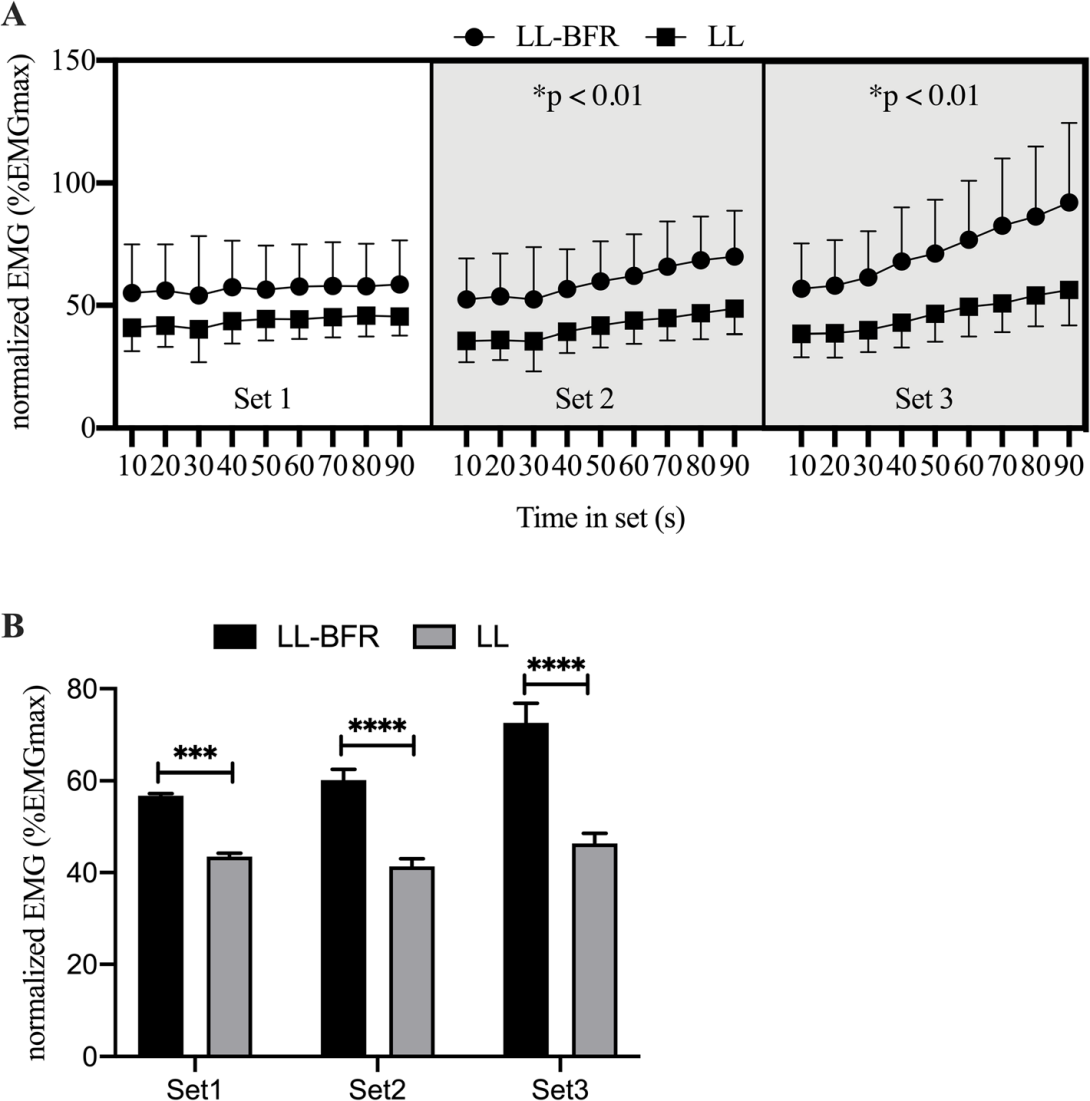
Change in muscle activation between LL-BFR (filled circle) and LL (filled squares) across the three sets (**a**). The data points show the EMG activity in 10 s intervals within each set. There was a significant higher EMG level in each of the sets in the BFR condition (**b**). Data are presented as mean and SEM. *** = *p* = 0.0005, **** = *p* < 0.0001

During MVC assessments, maximal EMG amplitude remained similar without a significant time × condition interaction (*p* > 0.05). During the three 90s sets, EMG amplitude significantly increased over time being significantly higher in the LL-BFR condition in set 2 (time x condition; *p* < 0.01; η_P_ = 0.253) and set 3 (time x condition; *p* < 0.01; η_P_ = 0.427: Fig. 1a). Furthermore, mean EMG activity during each of the three 90s sets was significantly higher in the LL-BFR condition in all sets (time x condition; *p* = 0.005; η_P_ = 0.377; Fig. 1b).

### Metabolic accumulation

**Fig. 2:**
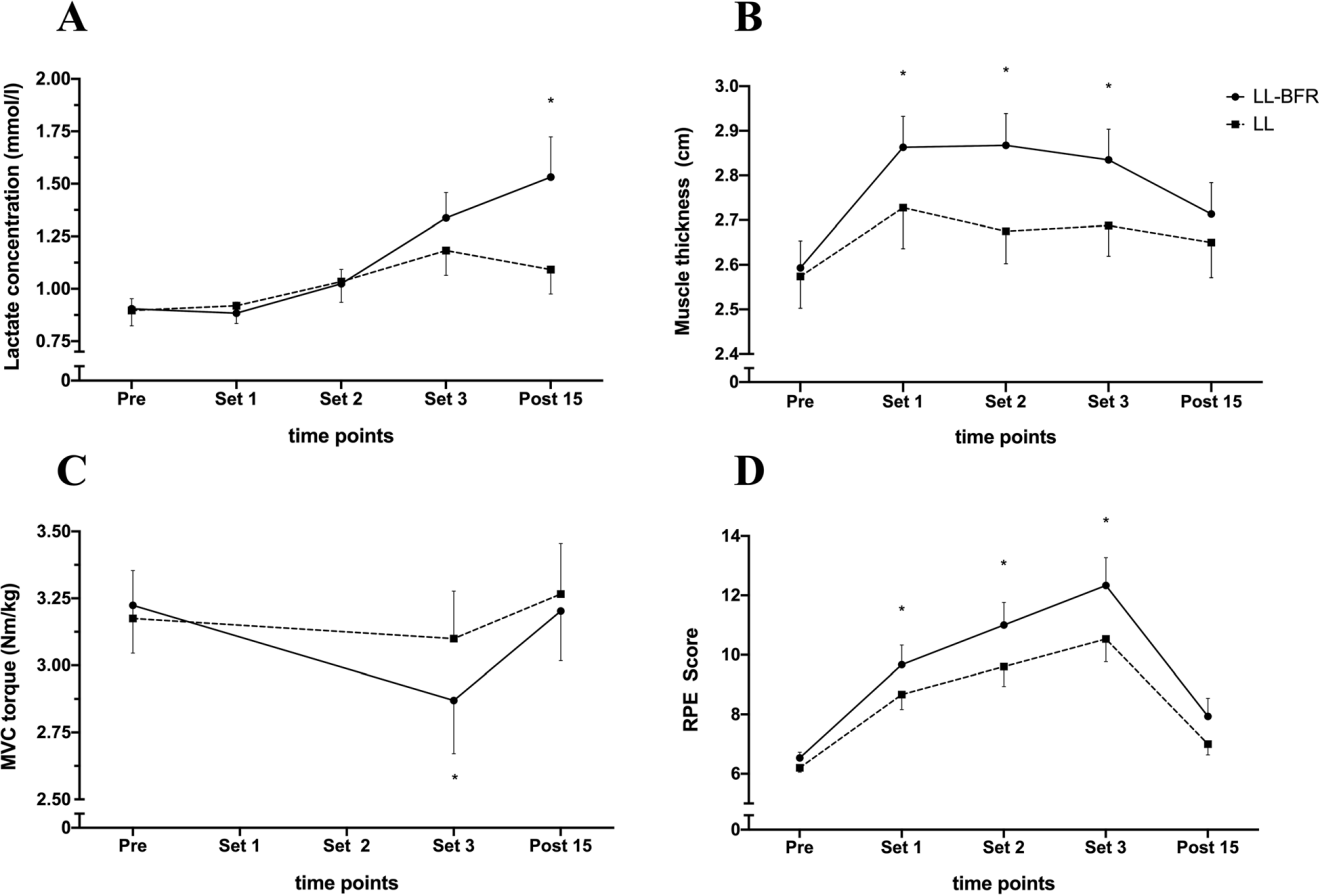
Changes in lactate concentration (**a**), muscle cell swelling (**b**), MVC (**c**) and RPE (**d**) between LL-BFR (filled circle) and LL (filled squares) across the three sets. Data are presented as mean and SEM. * = *p* < 0.05

Regarding metabolic stress, statistical analyses indicated a significant main effect of time (*p* < 0.01; η_P_ = 0.529) but no main effect of condition (*p* = 0.193; η_P_ = 0.118). However, a significant interaction effect (*p* < 0.01; η_P_ = 0.390) was observed with significantly higher increases in lactate concentrations in the LL-BFR condition 15 min following exercise (Fig. 2a). Although total lactate concentrations are still in a moderate range, lactate levels increased by 69% in the LL-BFR and only 22% in the LL condition.

### Muscle cell swelling

Evaluation of muscle thickness revealed a significant main effect of time (*p* < 0.01; η_P_ = 0.547) and condition (*p* < 0.01; η_P_ = 0.498). Moreover, a significant interaction effect (*p* < 0.05; η_P_ = 0.175) in favor of higher increases in muscle cell swelling in the LL-BFR condition was observed (Fig. 2b). These differences were statistically significant immediately following the three exercise sets (*p* < 0.05).

### Decrements in maximum voluntary torque

After statistical analysis, rmANOVA revealed a significant interaction effect (*p* < 0.05; η_P_ = 0.224) in favor of a greater post-exercise decrement in MVC torque in the LL-BFR condition. Additionally, a significant main effect of time (*p* < 0.05; η_P_ = 0.267) but not condition (*p* = 0.355; η_P_ = 0.061) was observed. MVC torque decreased from rest to post exercise from 3.22 Nm/kg to 2.87 Nm/kg in the LL-BFR condition and from 3.17 Nm/kg to 3.09 Nm/kg in the LL condition. After exercise, levels of MVC torque recovered to 3.20 Nm/kg and 3.26 Nm/kg in the LL-BFR and LL condition, respectively (Fig. 2c).

### Perceptual responses

The rmANOVA showed a significant main effect of time (*p* < 0.01; η_P_ = 0.655) and condition (*p* < 0.01; η_P_ = 0.449). Additionally, a significant time × condition interaction was observed (*p* < 0.05; η_P_ = 0.161) (Fig. 2d).

## Discussion

The current study aimed to obtain a holistic picture of potential physiological mechanisms underlying isometric low-load blood flow restriction training. Our findings demonstrate that the addition of blood flow restriction during isometric LL knee extensions augments muscle activation and facilitates the accumulation of metabolic stress. Additionally, LL-BFR induced significantly higher changes in muscle cell swelling and caused a significantly greater decline in MVC.

### Muscle activation

The results from this study revealed that the addition of BFR elicited significantly higher EMG amplitudes compared to the same exercise under free flow conditions. Previous investigations have primarily focused on the effects of dynamic exercises and found an enhanced neuromuscular activation following low-load dynamic exercises with BFR compared to LL alone [14–16]. Studies investigating the influence of an isometric contraction mode are scarce. Moritani and colleagues assessed EMG amplitude of the flexor carpi radialis-palmaris longus muscle during isometric contractions at 20% MVC with and without BFR. The authors found that when BFR was applied, EMG amplitude increased following LL-BFR compared to LL alone. Additionally, pronounced increases in motor unit spike amplitude and firing frequency were observed with BFR. This is in line with the augmented muscle excitability under LL-BFR conditions in the present study which was conducted in lower extremities (Fig. 1a+b). Similar increases in EMG have previously been reported under dynamic conditions [14, 16, 30] when BFR exercise was not performed until muscular fatigue [31] suggesting that muscle activity increases with LL-BFR under both dynamic as well as isometric conditions. One potential explanation might be that exercising with partial vascular occlusion results in increased motor unit spike amplitudes and firing frequencies [30]. This might be caused by an increased recruitment of fast-twitch muscle fibers [32] resulting in greater actions potentials [33]. This assumption is supported by finding from Krustrup and colleagues [32] who showed that after low-load knee extensor exercise with BFR, ~ 90% of the fast-twitch muscle fibers had decreased phosphocreatine levels resulting as a consequence of being active during the BFR contractions. Furthermore, in an explorative approach with *N* = 8 participants, Fatela and co-workers [33] used high-density EMG to non-invasively assess MU recruitment patterns and firing frequency. Their results also point towards an increased activity of MU with higher action potential amplitudes.

### Metabolic accumulation

Metabolic stress has been reported as being a primary contributor to the observed muscular adaptations (e.g. muscle hypertrophy) following LL-BFR training [6]. Previous in-vitro studies indicated that lactate stimulates early differentiation in C2C12 myoblasts and enhances p70S6K activity [34]. As a downstream target of the mammalian target of rapamycin (mTOR), the p70S6K complex has been identified as a key regulator of muscle protein synthesis [35]. However, these results have not been validated in vivo. Nevertheless, significant rises in lactate levels have been found following LL-BFR training in the present as well as other studies [7, 17] and could be explained by the induction of local hypoxia and the limited potential of lactate clearance during LL-BFR exercise [7]. Interestingly, most studies have again applied dynamic resistance exercise protocols and the effects of isometric muscle contractions remain unclear. In the present study, lactate levels during isometric contractions also increased after completion of the last exercise set compared to the LL condition under free blood flow conditions. One explanation that we did not see significant differences in lactate concentrations before the Post 15 test might be that the continuous application of the cuff prevented lactate clearance. This might have been the reason for non-detectable changes in systemic lactate concentrations before cuff deflation, which could explain the non-significant differences in lactate levels between LL-BFR and LL during the exercise. After deflation of the cuff, systematic lactate concentrations immediately increased supporting this theory. To examine this hypothesis, further studies are needed which assess metabolic accumulation on both systemic and local levels. Although a previous study found similar changes for local and systemic lactate concentrations after dynamic BFR exercise [10], these results might not necessarily be valid for isometric conditions.

### Cell swelling

Muscle cell swelling is a relatively novel factor within the mechanistic explanation for the observed BFR adaptations [6]. Nonetheless, several studies have examined the acute effects of BFR on cell swelling. Non-invasively, cell swelling was primarily assessed using ultrasound techniques [9] and it is well acknowledged that BFR training facilitates pronounced acute increases in muscle thickness [36]. Interestingly, the changes in muscle thickness in the present study were ~ 7% higher during LL-BFR than without (LL-BFR: 11%, LL: 4%). The physiologic explanation on how this phenomenon might impact muscle protein synthesis is believed to lay within the increased intra-muscular metabolic accumulation which favours fluid shifts into the intracellular space of the muscle fibres and thus induces structural stress on the cell membrane and a subsequent increase in a structure-positive signalling response [6].

### Decrements in maximal voluntary torque

Prolonged decrements in muscle torque following exercise would have a negative impact on muscle function and may be an indicator of excessive muscle damage and injury [37]. Within BFR research, declines in muscle torque following dynamic BFR training to volitional fatigue have been repeatedly reported in the scientific literature [38, 39]. Umbel and colleagues performed three sets of unilateral knee extensions to failure (35% MVC) and found that after 24 h, MVC was decreased by 14% in the LL-BFR condition with no significant decline (− 1.5%) in the LL condition [38]. In a second study by Sieljacks et al. [39] found numerically similar decrements in muscle torque following five sets of unilateral knee extensions at 30% 1RM to volitional failure. Interestingly, when the contractions were not performed until failure (four sets with 30–15–15-15 repetitions at 30% 1RM), these decrements in MVC quickly recovered to baseline levels [37]. This is in line with the present investigation which demonstrated an acute drop in torque of ~ 11% in the LL-BFR and ~ 3% in the LL condition (Fig. 2d). In both conditions, baseline levels of maximum voluntary torque recovered 15 min following the respective exercise session. Importantly, the significant reduction in knee extensor torque in the present study was seen even though subjects performed the isometric contraction with only 20% MVC being lower than the 30% applied in the study by Loenneke et al. [37]. This is of particular relevance for musculoskeletal rehabilitation and elite sport settings, where extensive muscle damage and concomitant inflammation is frequently contraindicated [40]. In this context, a quick restoration of muscle function is desired and therefore, a prolonged decrement in torque would be counterproductive.

### Perceptual responses

Recent studies have revealed that degree of perceived exertion might be associated with adherence rate [41]. Additionally, ratings of perceived exertion are frequently used in rehabilitation to monitor the rehabilitation program and load progression [42]. The present study showed that the rating of perceived exertion reached significantly higher scores following all three sets with LL-BFR compared to LL. However, these values return to baseline levels following 15 min after cessation of exercise. Previous trials, however, have demonstrated that perceptual responses following LL-BFR training are similar compared to high-load resistance training [10, 43]. Although the responses of the present study indicate higher RPE scores in the LL-BFR group, it has previously been revealed that these exacerbated perceptual responses subside after repeated BFR exercise sessions [43, 44]. It needs to be highlighted though, that high RPE values at lower loads can also be beneficial for example when athletes recover from injuries where BFR training would allow the athlete to maintain resiliency and have an outlet to exert high amounts of effort when high loads are contraindicated.

### Limitations

Unilateral isometric knee extension exercise was chosen in this study in order to evaluate the physiological mechanisms underlying isometric LL-BFR training in a standardized manner. Therefore, the results are not necessarily valid for bilateral or multi-joint dynamic exercises. Furthermore, we did not include a high load exercise group. Although, this was not necessary to answer the respective research question, further studies are needed which compare both LL-BFR and conventional high-load exercise regimens. Additionally, we have to make aware that only the acute decrements in MVC torque were assessed and potential further decrements in muscle function (e.g. 24 h or 72 h post exercise) were not evaluated. With respect to our results, we speculate that there are probably no serious changes, since already 15 min post exercise MVC torque levels were similar to baseline values. Lastly, these results were obtained from healthy and resistance-trained males and further studies are warranted to investigate the effects of isometric LL-BFR training in a clinical setting.

## Conclusion

The findings of the present study showed that muscle activation, metabolic accumulation, cell swelling as well as ratings of perceived exertion are increased following an isometric LL-BFR exercise at 20% MVC not performed to volitional fatigue. Although acute decrements in MVC torque were observed, these recovered within 15 min following training. Thus, it seems that isometric LL-BFR training might be a feasible and promising tool to facilitate muscular adaptations. Further research is, however, needed to validate these results in long-term trials and with clinical populations. Additionally, a direct comparison between dynamic and isometric LL-BFR training might be helpful to further elucidate potentially different adaptations.

## Data Availability

Add data are available on reasonable request from the corresponding author.

## Abbreviations

**1RM**: One repetition maximum
AOP: Arterial occlusion pressure
BFR: Blood flow restriction
EMG: Electromyography
GH: Growth Hormone
IL-4: Interleukin-4
IL-10: Interleukin-10
LL: Low load
LL-BFR: Low load blood flow restriction
MU: Motor unit
MVC: Maximum voluntary contraction
RF: Rectus femoris
RMS: Root mean square
RPE: ratings of perceived exertion
VL: vastus lateralis
